# Molecular Identification and Bioinformatics Analysis of *Anaplasma marginale* Moonlighting Proteins as Possible Antigenic Targets

**DOI:** 10.3390/pathogens13100845

**Published:** 2024-09-28

**Authors:** Rosa Estela Quiroz-Castañeda, Hugo Aguilar-Díaz, Eduardo Coronado-Villanueva, Diego Israel Catalán-Ochoa, Itzel Amaro-Estrada

**Affiliations:** Centro Nacional de Investigación Disciplinaria en Salud Animal e Inocuidad, Instituto Nacional de Investigaciones Forestales, Agrícolas y Pecuarias (CENID-SAI, INIFAP), Jiutepec 62574, México; aguilar.hugo@inifap.gob.mx (H.A.-D.); villanueva20032009@gmail.com (E.C.-V.); catalanochoaisrael@gmail.com (D.I.C.-O.); amaro.estrada@gmail.com (I.A.-E.)

**Keywords:** moonlighting proteins, pathogen, invasion, host, cell adhesion

## Abstract

Background: Diseases of veterinary importance, such as bovine Anaplasmosis, cause significant economic losses. Due to this, the study of various proteins of the causal agent *Anaplasma marginale* has focused on surface proteins. However, a vaccine for this disease is not yet available. To this end, in this work, moonlighting proteins (MLPs) are presented as an alternative approach for the design of immunogens against *A. marginale*. Methods: The proteins of the strain MEX-15-099-01 were analyzed, and its MLPs were identified. Subsequently, four virulence-associated MLP genes were selected and identified using PCR. The proteins were analyzed using a structural homology approach and the collection of B-cell epitopes was predicted for each MLP. Finally, a pair of AmEno peptides were synthesized and the antigenic potential was tested using an iELISA. Results: Our bioinformatics analysis revealed the potential of AmEno, AmGroEl, AmEF-Tu, and AmDnaK proteins as promising candidates for designing immunogens. The PCR allowed the gene sequence identification in the genome of the strain MEX-15-099-01. Notably, AmEno-derived synthetic peptides showed antigenicity in an ELISA. Conclusions: Our study has shed light on the potential use of MLPs for immunogen design, demonstrating the antigenic potential of AmEno.

## 1. Introduction

Tick vectors transmit *Anaplasma marginale*, the causal agent of bovine Anaplasmosis, an infectious disease affecting cattle in the world’s tropical regions and generating significant economic losses; additionally, mechanical transmission by biting insects and veterinary instruments can also occur [1]. This disease mainly affects yearlings and 2-year-old animals in a moderately severe way, while in older cattle, it is severe and often fatal; the most marked clinical signs are anemia, jaundice, weight and milk loss, and even death [2]. In the search for immunogenic targets that confer protection against Anaplasmosis, many *A. marginale* proteins have been studied, such as major surface proteins (MSPs), outer membrane proteins (OMPs), and type IV secretion system proteins [3,4,5]. Unfortunately, for decades, immunoprotective approaches against *A. marginale* have been unsuccessful [6,7].

Evolutionarily, the intracellular bacteria of the Order Rickettsiales, *A. marginale*, underwent a genome reduction, leading to a small size ranging from 0.8 to 2.5 Mbp [8]. This reductive evolution is positively associated with pathogenicity; therefore, proteins should be used efficiently [9]. In this regard, moonlighting proteins (MLPs) represent an evolutionary advantage that allows for the performance of multiple functions using the same genomic content [10]. Thus, the term MLPs refers to monomeric or multimeric proteins that perform numerous, autonomous, and unrelated functions independently in a different cellular localization [11,12,13]. MLPs do not include proteins with multiple functions resulting from different RNA splice variants, gene fusions, or several homologous proteins (isoforms), nor proteins with the same function in other locations or with different substrates [14]. Some canonical functions of MLPs include enzymes, scaffolds, ribosomal proteins, receptors, channels, chaperones, and housekeeping proteins associated with primary metabolism [13,15,16]. The secondary/moonlighting functions of MLPs include transcriptional regulation, receptor binding, apoptosis, and regulatory functions [17]. Thus, MLPs are considered molecular links of biological processes that facilitate the connection of different metabolic pathways (pathogenesis, cell division, apoptotic signaling, biosynthesis, and transport) by regulating or generating interactions between them [17,18].

Although many MLPs have been reported in several organisms (bacteria, archaea, yeast, fungi, humans, and others), advances in studying these proteins have never been addressed in *A. marginale*. However, the bioinformatics characterization of MLPs such as Enolase (Eno) from *A. marginale* was recently reported, as well as the role of MLPs in adhesion, plasminogen-binding, and probably in the host’s erythrocyte internalization [19,20,21,22,23,24]. Unfortunately, Eno has not been proposed as an immunological target against this pathogen. Thus, it is imperative to search for new strategies based on MLPs, taking advantage of their multifunctional activities, especially if we put into perspective the fruitless achievements in Anaplasmosis vaccine development [25].

In this work, we used bioinformatics tools to identify a vast repertoire of MLPs in seven Mexican strains of *A. marginale*. Later, we functionally annotated MLPs of the *A. marginale* strain MEX-15-099-01 and selected four proteins with reported evidence of their role as virulence factors, whose genes were confirmed using end-point PCR in *A. marginale* genome. In addition, we performed a structural homology analysis with the chosen MLPs Eno, GroEl, DnaK, and EF-Tu from pathogenic bacteria. Finally, we designed multiple antigenic peptides (MAPs) for *A. marginale* Eno and tested the antigenic potential by an indirect ELISA (iELISA).

This study reveals the potential of MLPs as possible candidates for prevention strategies and is a novel application of *A. marginale* proteins to control tick-borne diseases.

## 2. Materials and Methods

### 2.1. Genomic Identification of Moonlighting Proteins

For the identification of MLPs, firstly, the seven draft genomes of *A. marginale* Mexican strains previously reported [26,27,28] were automatically annotated with the RAST server (version 2.0) [29]. The NCBI reference sequence numbers of draft genomes were as follows: MEX-31-096-01 (NZ_VTWV00000000.1), MEX-30-193-01 (NZ_VTCZ00000000.1), MEX-30-184-02 (NZ_VTCY00000000.1), MEX-17-017- 01 (NZ_VTCX00000000.1), MEX-15-099-01 (VTWW01000001.1), MEX-14-010-01 (NZ_VTSO00000000.1), and MEX-01-001-01 (NZ_QLIV00000000.1). Then, the MLP identification was performed in the MoonProt 3.0 server, using the data retrieved from the RAST annotation of each *A. marginale* draft genome [30]. Subsequently, all MLPs identified in each genome were compared individually to evaluate whether the seven genomes share the same moonlighting proteins. Finally, for further analysis, we selected the *A. marginale* strain MEX-15-099-01 draft genome that contained the major number of MLPs and protein-encoding genes and rRNA and tRNA genes, according to MoonProt 3.0 and RAST server, respectively.

### 2.2. Identification of Moonlighting Genes by End-Point PCR

The molecular identification of four *A. marginale* genes encoding MLPs (AmEno, AmGroEl, AmDnaK, and AmEF-Tu) was performed with PCR using strain MEX-15-099-01 genomic DNA and specific primers to each gene sequence. The primers used were FwdEno: 5′-GTGCTAAGTGCTAGATCAGTTG-3′ and RevEno: 5′-TATCACATTATAAAGAAGAGC ACTC-3′; FwdGroEl: 5′-ATGGCGAATGTTGTTGTTACG-3′ and RevGroEl: 5′-TCCGCCCATTCCTCCCAT-3′; FwdDnak: 5′-ATGGCGGCTGAGCGCATC-3′ and RevDnak: 5′-CTACTTCTTGTCTTCGTCGTC-3′; and FwdEFTu: 5′-GTGAAAGACATAGTC ACATGG-3′ and RevEFTu: 5′-CTACTCCAAAATCTCAGTTATGA-3′. The PCR reaction was prepared with 12.5 μL of OneTaq^®^ 2X Master Mix with Standard Buffer (New England Biolabs, Ipswich, MA, USA), 1 μL of Forward primer (10 pmol/μL), 1 μL of Reverse primer (10 pmol/μL), 50 ng of genomic DNA, and MilliQ water to a final volume of 25 μL. For AmEno, the PCR condition was as follows: one cycle at 94 °C/5′, 30 cycles of 94 °C/3′, 60 °C/1′, 72 °C/1’20″, and a final cycle at 72 °C/5′. The condition for AmGroEl PCR amplification was one cycle at 94 °C/5′, 30 cycles of 94 °C/3′, 58 °C/1′, 72 °C/1′40″, and a final cycle at 72 °C/5′. As far as AmDnaK, the PCR condition was one cycle at 94 °C/5′, 30 cycles of 94 °C/3′, 59 °C/1′, 72 °C/1´56″, and a final cycle at 72 °C/5′. Finally, for AmEF-Tu, the condition used was one cycle at 94 °C/5′, 30 cycles of 94 °C/1′, 59 °C/1′, 72 °C/50″, and a final cycle at 72 °C/5′. The amplicons obtained were analyzed using electrophoresis on a 1% agarose gel at 100 V for 40 min and recorded in a UVP Bioimaging Systems EpiChemi 3 Darkroom Gel Imaging System (Richmond Scientific, Chorley, UK). The DNA molecular marker was 1 kb Plus DNA ladder N3200 (New England Biolabs). All amplicons obtained were gel-excised, purified (Wizard^®^ SV Gel and PCR Clean-Up System Quick, Promega, WI, USA), and sequenced at Unidad de Síntesis y Secuenciación de DNA, IBT-UNAM.

### 2.3. Functional Annotation

The genomic functional annotation of *A. marginale* strain MEX-15-099-01 was performed in Clusters of Orthologous Groups of proteins (COGs) in EGGNOG-mapper v2 (accesed on 21 January 2024, http://eggnog-mapper.embl.de/) [31].

### 2.4. Three-Dimensional (3D) Modelling

Based on their functional implications as virulence factors, we selected *A. marginale* strain MEX-15-099-01 MLPs (AmEno, AmGroEl, AmDnaK, and AmEF-Tu), associated with adhesion and invasion, and immunological evasion, as has been reported in other pathogen bacteria [32,33,34,35]. The 3D protein structures were modeled in the web-server SWISS-MODEL [36]. This server includes the AlphaFold Protein Structure Database (AlphaFold DB) [37] alongside the experimental structure-based templates from the SWISS-MODEL Template Library in the SWISS-MODEL homology-modelling pipeline. ChimeraX software [38] was used for the visualization and analysis of molecular structures in detail of 3D models generated in SWISS-MODEL.

### 2.5. Structural Homology Analysis

We performed a structural homology analysis to assess the potential role of AmEno, AmGroEl, AmDnaK, and AmEF-Tu as virulence factors. Thus, we selected the crystallized structure of *Streptococcus suis* Eno deposited in PDB (4EWJ), and the 3D structures predicted in AlphaFold DB for *Leptospira interrogans* GroEl (A0A4D8SCF3), *Mycoplasma hyorhinis* DnaK (K7XM09), and *Listeria monocytogenes* EF-Tu (A0A0Y7JXT9), and compared them with the 3D structures of selected MLPs. The superimposition of the molecular structures was performed and visualized in ChimeraX [38].

### 2.6. B-Cell Epitopes Prediction and Multiple Antigenic Peptides (MAPs) Design

The servers BcePred [39] and SVMTrip [40] were used to predict B-cell epitopes in *A. marginale* strain MEX-15-099-01 MLPs (AmEno, AmGroEl, AmDnaK, and AmEF-Tu). The BcePred server predicts B-cell epitopes based on the physicochemical properties of amino acids and SVMTrip performs realistic predictions of protein surface regions that are preferentially recognized by antibodies (antigenic epitopes). According to the functional characteristics, the antigenic and immunogenic capacity of Enolase reported in the literature, and its bioinformatic analysis, we chose the best predicted B-cell epitopes of AmEno. Then, two epitopes were used to design multiple antigenic peptides (MAPs), named AmEno1 and AmEno2. Both MAPs were structured by four repetitions of the same epitope, branched artificially to a Lysine residue scaffold, and synthesized at LifeTein^®^, Somerset, NJ, USA.

### 2.7. Indirect Enzyme-Linked ImmunoSorbent Assay (iELISA)

A total of 1 mg of each MAP was resuspended separately in 1 mL of MQ sterile water and diluted in 100 mL of 30 mM carbonate buffer (pH 9.5). Subsequently, 96-well microtiter plates (Corning, Tewksbury, MA, USA) were coated with 0.25 µg of each diluted MAP (100 µL per well) and incubated for 18 h at −20 °C. Then, plates were washed three times using 100 µL of phosphate-buffered saline (PBS), supplemented with 0.05% Tween 20 (PBS-T20) (Sigma-Aldrich, St. Louis, MO, USA) per well. Afterward, plates were blocked using 100 µL of 0.5% Bovine Serum Albumin (BSA, Sigma-Aldrich) for 2 h at 37 °C, and later, the plate was washed three times as mentioned above. After washing, plates were incubated with 100 µL containing different bovine sera diluted 1:100 in PBS-T20, and incubated for 1 h at 37 °C. Then, plates were washed as previously mentioned and incubated with anti-bovine IgG alkaline phosphatase-conjugated antibody (Sigma-Aldrich), diluted 1:30,000 in 0.05% PBS-T20 for 1 h at 37 °C. After incubation, the plate was washed thrice with 100 µL of 0.05% PBS-T20 and one wash with 100 mM Tris-base (pH 9.6). Later, the plate was incubated for 1 h at 37 °C with 100 µL of p-nitrophenyl phosphate substrate (SIGMAFAST, Sigma-Aldrich, St. Louis, MO, USA) and dissolved in buffer Tris-base (pH 9.6). Finally, the plates were read at 405 nm in a microtiter reader (MultiSkan FC, Thermo Scientific, Waltham, MA, USA). The control blank contained all components except serum, and all samples were run in triplicate. The mean absorbance of the blank control was used to normalize the absorbance of all samples. The cutoff value was calculated as the mean absorbance value plus three times the standard deviation of the negative serum. The positivity index value for each serum was determined as the ratio of the mean absorbance divided by the cutoff index value (where >1 = positive; <1 = negative).

## 3. Results

### 3.1. Genomic Identification of Moonlighting Proteins

**Figure 1:**
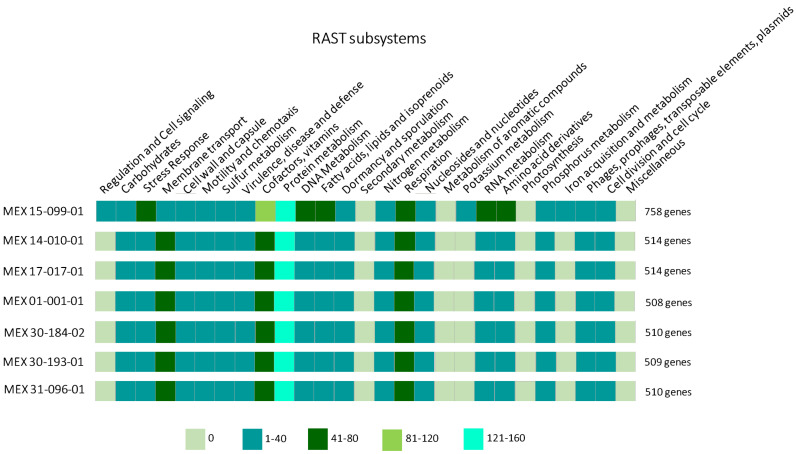
RAST subsystems classification of *A. marginale* Mexican strains. The strain MEX-15-099-01 has the most genes (758 protein-encoded genes and rRNA and tRNA genes) of all the strains. Additionally, protein metabolism is the subsystem with the most proteins and RNAs annotated. The five ranges of the gene numbers are shown at the bottom of the figure.

The genes number (protein-encoding genes and rRNA and tRNA genes) retrieved in the RAST server varied among Mexican strains of *A. marginale*. According to automatically annotated RAST data, the *A. marginale* strain MEX-15-099-01 was predicted with 758 protein-encoding genes and RNAs genes classified in 27 subsystems, whereas the number of annotated proteins in the rest of the strains ranged between 508 and 514 genes (Figure 1). Therefore, we used the strain MEX-15-099-01 for further analysis to identify MLPs.

Of the 758 proteins of the *A. marginale* strain MEX-15-099-01, the analysis in the MoonProt 3.0 database revealed a match with 80 MLPs, each one with two functions assigned, including a primary function (Function 1) and moonlighting function (Function 2) (Table 1). According to MoonProt 3.0, both functions have experimental biochemical and/or biophysical data, which provide robust evidence for MLP identification. Interestingly, we found that all MLPs of the Mexican strains were contained in the strain MEX-15-099-01, a novel and intriguing finding that opens up new avenues for research.

### 3.2. Identification of Moonlighting Proteins 

**Figure 2:**
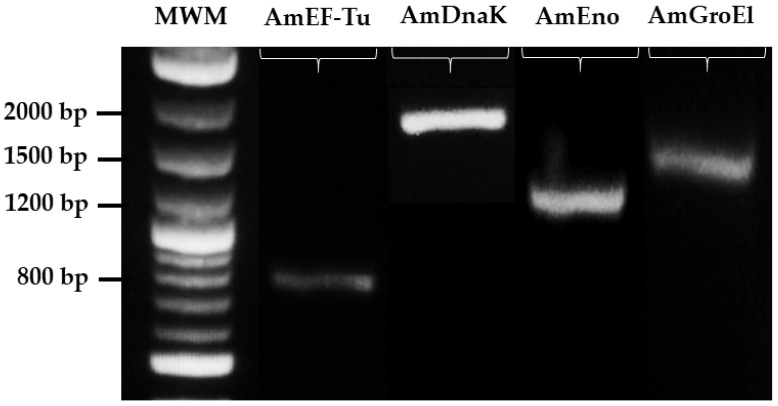
Molecular identification of *A. marginale* MEX-15-099-01 MLPs genes. Agarose gel electrophoresis showing PCR amplicons of AmEF-Tu (807 bp), AmDnaK (1926 bp), AmEno (1278 bp), and AmGroEl (1650 bp). Arrows point to the band size of the molecular weight marker (MWM).

The MLP gene identification of AmEno, AmGroEl, AmDnaK, and AmEF-Tu in the *A. marginale* genome was confirmed using the sequencing and amplicon size in the electrophoresis gel that corresponded to 1278 bp, 1650 bp, 1026 bp, and 807 bp, respectively (Figure 2).

### 3.3. Functional Annotation of MLPs

**Figure 3:**
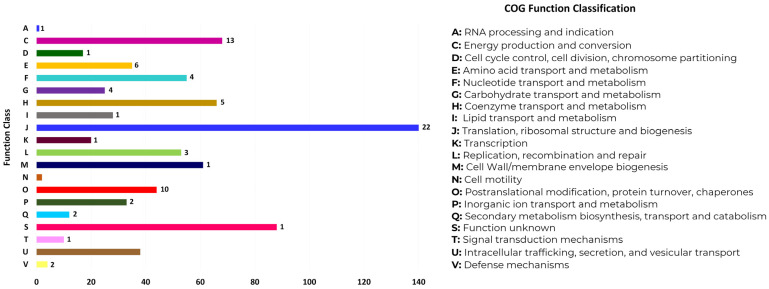
COG function classification of *A. marginale* strain MEX-15-099-01 proteins. The 80 MLPs are distributed in 18 out of 20 different functional groups. Categories C (energy production and conversion), J (translation, ribosomal structure, and biogenesis), and O (posttranslational modification, protein turnover, and chaperones) comprise the majority of MLPs. Each functional group is shown in the right panel, and the number of MLPs identified in each functional group is shown in bold numbers on one side of the bar.

**Figure 4:**
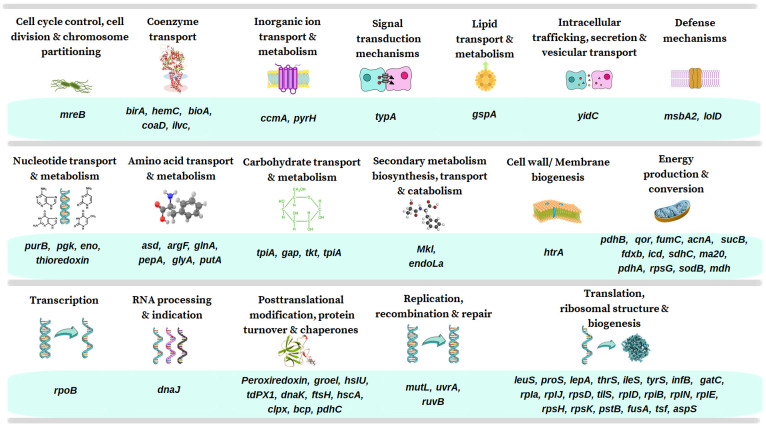
Identification of MLPs genes in *A. marginale* strain MEX-15-099-01. MLPs genes are shown in the 18 functional groups: energy production and conversion, translation, ribosomal structure and biogenesis, and posttranslational modification, protein turnover, and chaperones grouped the major number of genes.

We performed the functional annotation of the *A. marginale* strain MEX-15-099-01 MLPs in the COG database, which classifies proteins based on the orthologs (direct evolutionary counterparts). The COG function classification showed that all proteins of the *A. marginale* strain MEX-15-099-01 were classified into 20 functional groups, and specifically, 18 of them contained the MLPs of the *A. marginale* strain MEX-15-099-01 (Figure 3 and Figure 4 and Supplementary Table S1).

### 3.4. Three-Dimensional (3D) Modelling

To ensure the utmost accuracy in our research, the process of obtaining the 3D structures of AmEno, AmGroEl, AmDnaK, and AmEF-Tu was guided by the selection of templates with the highest Global Model Quality Estimation value (GMQE) in SwissModel. For AmEno, the best template according to GMQE 0.96 was the *A. marginale* strain St. Maries Eno (AlphaFold DB accession number Q5PAS6), which shares a sequence identity of 99.53% and a coverage of 100%. The template for AmEF-Tu, with GMQE 0.92, was the *Erlichia ruminatum* strain Gardel EF-Tu (Q5FFE6), with a sequence identity of 89.57% and coverage of 100%. Regarding AmGroEl, the template with the best GMQE 0.88 was *A. marginale* GroEl (Q84I74), with an identity and coverage of 99.64% and 99.64%, respectively. Lastly, the best template for obtaining the 3D model of AmDnaK was *Wolbachia pipientis* (Q73GL7), with a GMQE of 0.87, sequence identity of 71.66%, and coverage of 88%.

### 3.5. Structural Homology Analysis

**Figure 5:**
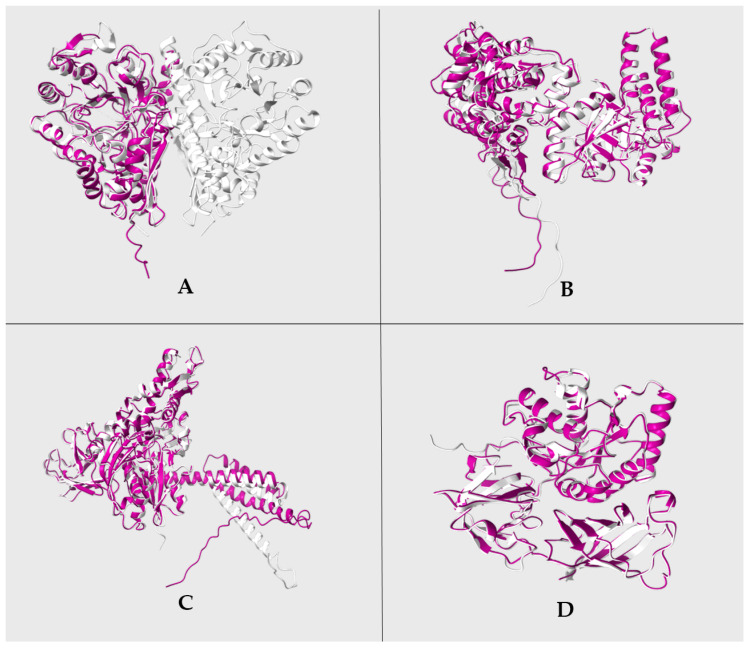
Structural homology of *A. marginale* strain MEX-15-099-01 MLPs. Superimpositions of (**A**) AmEno (magenta) and *S. suis* Eno (silver); (**B**) AmGroEl (magenta) and *L. interrogans* GroEl (silver); (**C**) AmDnaK (magenta) and *M. hyorhinis* DnaK (silver); (**D**) AmEf-Tu (magenta) and *L. monocytogenes* EF-Tu (silver). RMSD values obtained for each superimposition were 0.682 Å, 0.939 Å, 0.644 Å, and 0.455 Å, respectively.

To analyze if AmEno, AmGroEl, AmDnaK, and AmEF-Tu 3D models share structural homology with moonlighting functions in adhesion, invasion, and immune evasion, we performed a 3D superimposition with *S. suis* Eno, *L. interrogans* GroEl, *M. hyorhinis* DnaK, and *L. monocytogenes* EF-Tu, and assessed the Root Mean Square Deviation (RMSD) value. The results showed that AmEno, AmGroEl, AmDnaK, and AmEF-Tu 3D superimposition had low RMSD values, indicating low variability between 3D structures. In this regard, RMSD values are considered reliable indicators of variability when applied to similar proteins; a good RMSD is ≤2.0 Å, an acceptable RMSD is >2.0 Å and <3.0 Å, and a bad RMSD is ≥3.0 Å. A value of RMSD 0 corresponds to identical structures, which suggests a significant structural identity. Interestingly, we observed an RMSD of 0.939 Å between AmGroEl and *L. interrogans* GroEl, and a low RMSD value of 0.682 Å was obtained in the structural superimposition of AmEno and *S. suis* Eno, suggesting a significant structural identity among these proteins. Even lower RMSD values were obtained in the superimpositions of AmDnaK and *M. hyorhinis* DnaK, and AmEF-Tu and *L. monocytogenes* EF-Tu, with a RMSD of 0.644 Å and 0.455 Å, respectively (Figure 5).

### 3.6. B-Cell Epitope Prediction

The epitope-based in silico approach is immunologically relevant since small sequences often induce protective immunity against pathogens. In this regard, we presented a collection of B-cell epitopes with predicted antigenic potential for *A. marginale* MLPs (AmEno, AmGroEl, AmDnaK, and AmEF-Tu) (Table 2).

### 3.7. Antigenic Potential of AmEno1 and AmEno2

**Figure 6:**
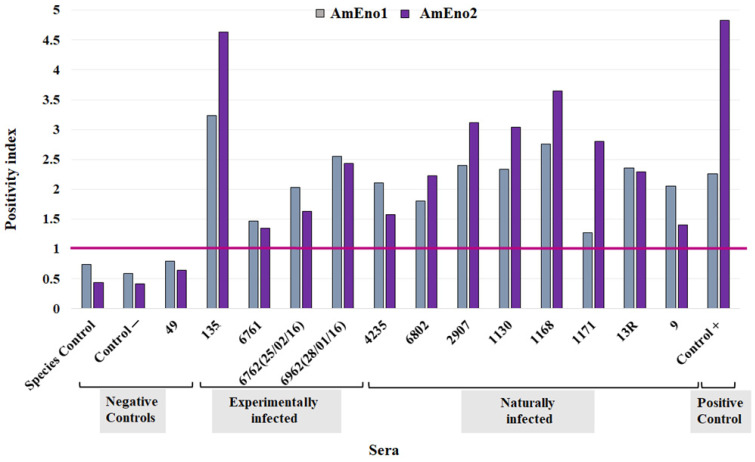
Antigenic potential of multiple antigenic peptides. Positivity index values obtained using *A. marginale* AmEno1 and AmEno2 and sera from experimentally and naturally infected animals. Sheep serum (species control); sera from hyperimmunized animal (135). The horizontal magenta line indicates the cutoff value, where values ≥ 1 are positive and values < 1 arenegative.

The iELISA using 0.25 µg of MAPs AmEno1 and AmEno2 showed that peptides have an antigenic potential. The antibodies in the sera from animals naturally and experimentally infected with *A. marginale* reacted against both peptides with positivity indexes exceeding 1. In naturally infected animals, serum 1168 showed the highest positivity values, at 2.75 (AmEno1) and 3.65 (AmEno2) (Figure 6). In experimentally infected animals, the highest positivity values are those of serum 135, with 3.24 (AmEno1) and 4.63 (AmEno2). It is noteworthy that this serum was from a hyperimmunized animal with *A. marginale*. These results showed that AmEno1 and AmEno2 were recognized by the specific antibodies against *A. marginale*.

## 4. Discussion

MLPs are distributed in diverse organisms from the three domains of life. These proteins mainly participate in primary metabolic processes and show additional secondary functions. Interestingly, many of these functions in pathogens are associated with pathogenesis and survival in a host [23,39]. In this regard, in bacterial and fungal pathogens, MLPs participate in the adhesion, invasion, and colonization of mucosal surfaces or even act as toxins; thus, a genuine interest exists in understanding their roles as virulence factors [23,40].

Many MLPs identified in pathogenic bacteria can bind to specific components of the extracellular cell matrix (fibronectin, laminin, elastin, and collagen) [19,41,42]. For instance, Eno from *Staphylococcus aureus* has been shown to bind to laminin and collagen I, and EF-Tu of *Pseudomonas aeruginosa* bind to host complement system factors and plasminogen [43,44]. These findings underscore the potential of MLPs as vaccine candidates. On the other hand, the study of *A. marginale* surface proteins (major surface proteins and outer membrane proteins) as a primary strategy to control bovine anaplasmosis has not been successful [45,46]. However, the study of *A. marginale* MLPs presents an intriguing opportunity to contribute to understanding the mechanisms associated with pathogenesis. For instance, bioinformatics analyses of MLPs suggest that AmEno could bind to proteins on the red blood cell surface, highlighting its potential role in adhesion and possibly in the bacteria internalization, processes still uncharacterized in this pathogen [24].

Thus, in this work, we identified a collection of MLPs in *A. marginale* Mexican strains and performed a structural homology analysis to determine their possible function compared to previously reported MLPs in pathogenic bacteria. Firstly, to cover the major number of proteins from Mexican strains, we selected strain MEX-15-099-01, which contains 758 protein-encoding genes and rRNA and tRNA, according to RAST annotation, and then we identified MLPs in the MoonProt 3.0 database. We found 80 MLPs that were mainly related to functions of primary metabolism, and the second function is related to signaling or regulating processes such as transcription or translation, as expected [47]. Of these 80 MLPs, we selected four MLPs (AmEno, AmGroEl, AmDnaK, and AmEF-Tu) with a significant role in virulence processes, and, as it was expected, the MLP genes were identified in the *A. marginale* genome.

Interestingly, some identified MLPs had a second function related to virulence factors, such as AmGroEl and AmDnaK from category O (posttranslational modification, protein turnover, and chaperones), AmEF-Tu from category J (translation, ribosomal structure, and biogenesis), and AmEno from category F (nucleotide transport and metabolism). Specifically, GroEl and DnaK participate in proteasome assembly, transport, and the folding of proteins [48,49,50], and during pathogen invasion, they secrete or relocate to the cell surface to bind to plasminogen and mucin as a moonlight function [51,52,53]. For example, in *Mycoplasma pneumonia* and *Salmonella enterica,* GroEl relocates to the bacteria surface to promote adhesion to the glycoprotein of mucosal surfaces (mucin), allowing pathogen invasion and colonization [51,52,53]. Additionally, GroEl provides protective immunity in infections with *Mycoplasma tuberculosis*, *Salmonella typhimurium*, and *Streptococcus pneumonia* when it is used as a recombinant protein [52,54,55]. Conversely, DnaK is considered the most conserved member of the ubiquitous heat-shock protein 70 family of molecular chaperones [9]. In the pathogen of pigs, *Mycoplasma hyorhinis*, the recombinant DnaK, in addition to binding to plasminogen, also binds to fibronectin, laminin, type IV collagen, and vitronectin [34]. In contrast, in *M. tuberculosis*, recombinant DnaK binds human plasminogen and stimulates monocyte chemokine synthesis and dendritic cell maturation [56]. On the other hand, the G protein EF-Tu is a member of the category of translation, ribosomal structure, and biogenesis, which participates in protein folding and the catalysis of aminoacyl-tRNA to the A-site of the ribosome [57]. EF-Tu binds to fibronectin, plasminogen, and other extracellular matrix proteins in pathogenic bacteria, increasing virulence [58]. In pigs, the immunization with *S. suis* recombinant EF-Tu (rEF-Tu) elicited a Th1 and Th2 protective response against the bacteria, and the anti-rEF-Tu sera reduced the pathogen load in porcine blood [59]. Additionally, a proteomics analysis in a Brazilian isolate of *A. marginale* revealed that a group of proteins associated with the cell membrane, including EF-Tu, induced an IgG2-type immune response in immunized cattle with pathogen membrane fractions, suggesting a possible role of EF-Tu in antibody activation [60]. We have identified Eno or phosphopyruvate hydratase in the category of nucleotide transport and metabolism. This enzyme catalyzes the glycolytic reaction from 2-phosphoglycerate (2-PGA) to phosphoenolpyruvic acid (PEP). Eno is displayed on the cell surface of *Streptococcus pneumoniae,* where it binds to plasminogen and then converts to plasmin, a critical step in breaking down the extracellular matrix and initiating the invasion of host tissue [61]. Also, Eno was recently found to bind to the complement protein C4b-binding protein (C4BP) and protect bacteria from complement-mediated killing [56]. Furthermore, *S. suis* and *Staphylococcus aureus* Eno can bind to host fibronectin or laminin, respectively, and even to porcine red blood cells like *Mycoplasma suis* [22,32]. In this regard, in the bovine hemoparasite *Babesia bigemina,* an Eno was identified from an expressed sequence tags (ESTs) analysis of the intraerythrocytic stage, suggesting a possible role in the erythrocyte invasion [62]. More recently, Cárdenas-Flores et al. [63] found that the sera from rabbits immunized with multiple antigenic peptides (MAPs) derived from *Babesia bovis* Eno recognized the parasite by indirect immunofluorescence in infected bovine erythrocytes. This finding supports the possible location of Eno in the pathogen’s cell membrane or its interaction with red blood cells. In an immunogenicity study in pigs, Xue et al. [64] used a recombinant *M. suis* Eno (rMseno) that can induce an immunological response and partial clinical protection against the *M. suis* challenge. This immunogenicity makes rMseno a potential immunogen for developing an anti-*M. suis* vaccine, demonstrating the practical implications of this research.

Our structural homology analysis revealed that the selected *A. marginale* MLPs share a similar 3D structure with MLPs of pathogenic bacteria, acting as virulence factors in adhesion, invasion, and immune evasion [33,34,35,64]. This result suggested that *A. marginale* MLPs could have a similar function, making them potential vaccine candidates.

Therefore, in this work, we explored whether MAPs from AmEno could have antigenic potential. In this regard, our results revealed that positive sera to *A. marginale* recognized AmEno1 and AmEno2, which represent a good approach for using them as vaccine antigens. These results led us to continue working on the assessment of the antigenicity of the predicted B-cell epitopes, which could be the basis for developing vaccines against Anaplasmosis.

## 5. Conclusions

In recent years, the development of vaccine candidates against bovine Anaplasmosis has focused on cell surface proteins, the potential of which as such has not been successful so far. On the other hand, MLPs involved in different processes represent a viable alternative for controlling tick-borne diseases. In this regard, we are currently directing our efforts to explore MLPs, the study of which in other pathogens represents a possibility of developing control strategies based on multiepitope vaccines. In this work, we identified the MLPs of *A. marginale* and selected four that were associated with virulence factors. We studied these using bioinformatics tools with the aim of proposing these proteins as potential multiepitope vaccines. Additionally, we identified four MLP genes in the *A. marginale* genome, and finally, we designed MAPs derived from AmEno, which showed an antigenic potential. These bioinformatics approaches represent a feasible alternative for vaccine development, reducing time and costs in the search for strategies to control pathogens.

## Figures and Tables

**Table 1:** *A. marginale* strain MEX-15-099-01 proteins predicted as moonlighting in MoonProt 3.0 database.

	NCBI ID	*A. marginale* Strain MEX-15-099-01 Proteins	Protein Name and Species in MoonProt 3.0 Database	Function 1	Function 2	E-Value
1	KAA8473050.1	DNA mismatch repair endonuclease MutL	Mismatch repair endonuclease PMS2, *Mus musculus*	PMS2 mismatch repair enzyme introduces single-stranded breaks near the mismatch	Hypermutation of antibody variable chains	1.43 × 10^−28^
2	KAA8473081.1	ABC transporter ATP-binding protein	MalK, *Escherichia coli*	ATP binding/hydrolysis protein of MalEFGK maltose/maltodextrin transporter (importer), an ABC transporter ATP + H_2_O = ADP + phosphate	Transcription regulation binds to MalT activator of mal regulon and prevents its action	2.73 × 10^−48^
3	KAA8473084.1	Biotin-[acetyl-CoA-carboxylase] ligase	birA biotin sythetase, *Escherichia coli*	birA biotin synthetase, enzyme biotin-acetyl-CoA-carboxylase] ligase ATP + biotin + apo-[acetyl-CoA:carbon-dioxide ligase (ADP-forming)] = AMP + diphosphate + [acetyl-CoA:carbon-dioxide ligase (ADP-forming)]	Bio-operon repressor activity depends on cellular concentration of biotin	6.49 × 10^−19^
4	KAA8473103.1	Porphobilinogen synthase	Delta-aminolevulinic acid dehydratase, *Homo sapiens*	5-aminolaevulinate dehydratase, enzyme converts 2 (5-aminolevulinate) to porphobilinogen + 2H_2_O. Porphyrin-containing compound metabolism, protoporphyrin-IX biosynthesis	Proteasome inhibitor noncompetitively blocks proteolysis of certain protein substrates	1.14 × 10^−56^
5	KAA8473122.1	Leucine-tRNA ligase	Leucyl-tRNA synthetase, *Saccharomyces cerevisiae*	Leucyl-tRNA synthetase, enzyme ATP + L-leucine + tRNA (Leu) ⇒ AMP + diphosphate + L-leucyl-tRNA (Leu) protein synthesis	Intron splicing, RNA splicing group I intron splicing	1.53 × 10^−14^
6	KAA8473129.1	excinuclease ABC subunit UvrA	MDR1, *Homo sapiens*	Transmembrane transporter efflux pump, uses ATP for energy expels drugs and other small molecule compounds ATP + H_2_O + xenobiotic (Inside) => ADP + phosphate + xenobiotic (Outside)	Regulator of volume-activated chloride channels	2.23 × 10^−6^
7	KAA8473164.1	Proline-tRNA ligase	Threonyl-tRNA synthetase, *Escherichia coli*	Threonine-tRNA ligase, enzyme ATP + L-threonine + tRNA (Thr) => AMP + diphosphate + L-threonyl-tRNA (Thr)	Binds mRNA encoding threonyl-tRNA synthetase, controls expression of its own gene at the translational level	6.82 × 10^−12^
8	KAA8473168.1	Alkyl hydroperoxide reductase subunit C-like protein	Mitochondrial 2-cysteine peroxiredoxin *Leishmania infantum*	Peroxidase activity, detoxification of reactive oxygen species (ROS), removal of peroxide, uses redox active cysteine residue (peroxidatic Cys) to reduce substrates like H_2_O_2_	Chaperone and activators of signal transduction cascades, prevents thermal aggregation of citrate synthase in vitro, lack of expression makes promastigotes more sensitive to temperature in the mammalian host (37 °C)	1.27 × 10^−52^
9	KAA8473177.1	Translation elongation factor LepA	Elongation factor 2, *Homo sapiens*	Translation elongation factor	Binding partner for Akt2 signaling molecule	6.10 × 10^−24^
10	KAA8473193.1	Triosephosphate isomerase	Triose phosphate isomerase, *Staphylococcus aureus*	Triose phosphate isomerase, enzyme D-glyceraldehyde 3-phosphate ⇔ dihydroxyacetone phosphate Carbohydrate degradation, glycolysis Carbohydrate biosynthesis, gluconeogenesis	Adhesin, contact-mediated killing of Cryptococcus	4.52 × 10^−45^
11	KAA8473199.1	Adenylosuccinate lyase	Argininosuccinate lyase, *Anas platyrhynchos*	Argininosuccinate lyase, enzyme Catalyzes the breakdown of argininosuccinate to produce arginine and fumarate. It is the fourth enzyme of the urea cycle. Argininosuccinase is involved in biosythesis of arginine in all species and production of urea in ureotelic organisms. 2-(N(omega)-L-arginino)succinate => fumarate + L-arginine Amino-acid biosynthesis, arginine biosynthesis	Delta-2 Crystallin in the lens of the eye—only in birds and reptiles	6.71 × 10^−7^
12	KAA8473204.1	Aspartate aminotransferase	MalY, *Escherichia coli*	Beta-cystathionase, enzyme cleavage of cystathionine to homocysteine, ammonia, and pyruvate L-cystathionine + H_2_O => L-homocysteine + NH_3_ + pyruvate Amino-acid biosynthesis, methionine biosynthesis	Transcription regulation binds to MalT activator of mal regulon and prevents its action	1.42 × 10^−7^
13	KAA8473206.1	Rod shape-determining protein	DnaK, *Lactococcus lactis*	Chaperone	Binding to invertase, a hyperglycosylated mannoprotein from Saccharomyces cerevisiae	3.82 × 10^−9^
14	KAA8472839.1	Phosphopantothenoylcysteine decarboxylase	Phosphopantothenoylcysteine decarboxylase, *Schizosaccharomyces pombe*	Phosphopantothenoylcysteine decarboxylase, decarboxylation of phosphopahtothenoyl-L-cysteine, in CoA biosynthesis, PPCDC	Inhibitor of serine/threoinine phosphatase Pzh1	7.40 × 10^−25^
15	KAA8472842.1	Phosphoglycerate kinase	Phosphoglycerate kinase, *Streptococcus anginosus* and *S. oralis*	Phosphoglycerate kinase, enzyme ADP + 1,3-bisphosphoglycerate => ATP + 3-phospho-D-glycerate Carbohydrate degradation, glycolysis	Plasminogen binding	2.25 × 10^−81^
16	KAA8472854.1	Ketol-acid reductoisomerase	Acetohydroxyacid isomerase, *Saccharomyces cerevisiae*	Acetohydroxyacid reductoisomerase, enzyme Amino-acid biosynthesis, L-leucine, L-isoleucine and L-valine biosynthesis (R)-2,3-dihydroxy-3-methylbutanoate + NADP^+^ => (S)-2-hydroxy-2-methyl-3-oxobutanoate + NADPH (2R,3R)-2,3-dihydroxy-3-methylpentanoate + NADP+ => (S)-2-hydroxy-2-ethyl-3-oxobutanoate + NADPH Cofactor	maintain mitochondrial DNA stability enzyme catalytic Function not needed for this role	1.24 × 10^−25^
17	KAA8472865.1	Molecular chaperone DnaJ	Zuotin, *Saccharomyces cerevisiae*	Component of a chaperone complex component of the ribosome-associated complex (RAC) that helps in folding of nascent polypeptide chains	Activator of a transcription factor activates Pdr1 transcription factor	1.46 × 10^−6^
18	KAA8472874.1	NAD-dependent glyceraldehyde-3-phosphate dehydrogenase	GAPDH, *Bacillus anthracis*	Glyceraldehyde 3-phosphate dehydrogenase, enzyme catalyzes the oxidative phosphorylation of D-glyceraldehyde- 3-phosphate (G-3-P) to 1,3-diphosphoglycerate in the presence of NAD^+^/NADP^+^ and inorganic phosphate (Pi)	Plasminogen binding	2.06 × 10^−99^
19	KAA8472877.1	Aspartate-semialdehyde dehydrogenase	Arg5,6, *Saccharomyces cerevisiae*	Enzyme in the arginine biosynthetic pathway N-acetyl-gamma phosphate reductase and acetyl glutamate kinase	Arg5 binds to mitochondrial and nuclear DNA in vivo and regulates gene expression, regulator of transcription for several genes	1.16 × 10^−6^
20	KAA8472881.1	Threonine-tRNA ligase	Threonyl-tRNA synthetase, *Escherichia coli*	Threonine-tRNA ligase, enzyme ATP + L-threonine + tRNA(Thr) => AMP + diphosphate + L-threonyl-tRNA(Thr)	Binds mRNA binds mRNA encoding threonyl-tRNA synthetase, controls expression of its own gene at the translational level	0
21	KAA8472917.1	Ornithine carbamoyltransferase	Ornithine carbamoyltransferase, *Staphylococcus epidermidis*	Ornithine carbamoyltransferase, enzyme Carbamoyl phosphate + L-ornithine => phosphate + L-citrulline	Bind fibronectin	2.49 × 10^−16^
22	KAA8472922.1	ATP-binding cassette domain-containing protein	MalK, *Escherichia coli*	ATP binding/hydrolysis protein of MalEFGK maltose/maltodextrin transporter (importer) an ABC transporter ATP + H_2_O = ADP + phosphate	Transcription regulation binds to MalT activator of mal regulon and prevents its action	2.98 × 10^−23^
23	KAA8472925.1	Glutamine synthetase	Glutamine synthetase, *Bifidobacterium*	Glutamine synthetase, enzyme ATP + L-glutamate + NH_3_ => ADP + phosphate + L-glutamine	Plasminogen binding	2.53 × 10^−140^
24	KAA8472933.1	Pyruvate dehydrogenase complex dihydrolipoamide acetyltransferase	Pyruvate dehydrogenase E2 subunit, *Bacillus thuringiensis*	Pyruvate dehydrogenase E2 subunit, dihydrolipoamide acetyltransferase	DNA binding, transcription regulation	3.95 × 10^−54^
25	KAA8472947.1	Valine-tRNA ligase	Leucyl-tRNA synthetase, *Saccharomyces cerevisiae*	Leucyl-tRNA synthetase, enzyme ATP + L-leucine + tRNA (Leu) => AMP + diphosphate + L-leucyl-tRNA (Leu) protein synthesis	Intron splicing, RNA splicing group I intron splicing	1.53 × 10^−14^
26	KAA8472967.1	Tyrosine-tRNA ligase	Tyrosyl tRNA synthetase, *Neurospora crassa*	Tyrosyl tRNA synthetase, enzyme attaches tyrosine to tRNA (Tyr) ATP + L-tyrosine + tRNA (Tyr) = AMP + diphosphate + L-tyrosyl-tRNA(Tyr)	Promotes folding of group 1 introns	1.46 × 10^−56^
27	KAA8472719.1	Quinone oxidoreductase	NADPH quinone oxidoreductase, Zeta-crystallin, *Hyla japonica*	NADPH:quinone oxidoreductase, enzyme	Zeta crystallin (Also in camel, llamas and Guinea pig)	5.67 × 10^−29^
28	KAA8472720.1	Type I secretion system permease/ATPase	MDR1, *Homo sapiens*	Transmembrane transporter efflux pump, uses ATP for energy expels drugs and other small molecule compounds ATP + H_2_O + xenobiotic (Inside) => ADP + phosphate + xenobiotic (Outside)	Regulator of volume-activated chloride channels	1.96 × 10^−32^
29	KAA8472725.1	Class II fumarate hydratase	Aspartate ammonia lyase, aspartase, *Haemophilus influenzae*	Aspartate ammonia lyase, aspartase, enzyme L-aspartate => fumarate + NH_3_	Binds plasminogen	1.48 × 10^−101^
30	KAA8472731.1	Chaperonin GroEL	Hsp60, *Helicobacter pylori*	Chaperonin, prevents protein misfolding, promotes the refolding and proper assembly of unfolded proteins	Adhesin—to host cells	6.77 × 10^−169^
31	KAA8472736.1	Cytosol aminopeptidase PepA	PepA, *Escherichia coli*	Aminopeptidase, enzyme removes amino-terminal amino acid, preferentially if it is Leu	Transcriptional repressor binds DNA, binds car operator DNA represses the carAB operon	1.55 × 10^−84^
32	KAA8472754.1	NAD(P)H-dependent glycerol-3-phosphate dehydrogenase	Glycerol 3-phosphate dehydrogenase, *Candida albicans*	Glycerol 3-phosphate dehydrogenase, functions in glycerol accumulation	Plasminogen binding	1.38 × 10^−18^
33	KAA8472760.1	ATP-dependent protease ATPase subunit HslU	FtsH, *Shigella flexneri*	Chaperone	Metalloprotease, enzyme ATP-dependent zinc metallopeptidase, hydrolyzes cytoplasmic and transmembrane proteins	1.3 × 10^−6^
34	KAA8472784.1	Aconitate hydratase AcnA	Aconitase, *Bos taurus*	Aconitase, enzyme 4Fe-4S cluster in active site when cellular iron levels are high Citrate <=> isocitrate Citric acid cycle	Iron-responsive element binding protein when cellular iron concentrations are low, loses 4Fe-4S cluster and binds to iron-responsive elements (IRES) in mRNA that encodes proteins that are involved in iron uptake and use	0
35	KAA8472802.1	Methionine-tRNA ligase	Methionyl-tRNA synthetase, *Homo sapiens*	Methionyl-tRNA synthetase, enzyme ATP + L-methionine + tRNA(Met) => AMP + diphosphate + L-methionyl-tRNA(Met) protein synthesis	Biogenesis of rRNA in nucleoli translocation to nucleolus triggered by growth factors	4.55 × 10^−40^
36	KAA8472628.1	Superoxide dismutase	Superoxide dismutase, *Mycobacterium avium*	Superoxide dismutase, enzyme antioxidant converts superoxide anion radicals into O_2_ and H_2_O_2_	Adhesin	2.06 × 10^−31^
37	KAA8472646.1	Molecular chaperone DnaK	DnaK, Bifidobacterium(*Bifidobacterium lactis*, *B. bifidum*, and *B. longum*)	Chaperone	Plasminogen binding	0
38	KAA8472652.1	Transketolase	Transketolase, *Escherichi coli*	Transketolase, enzyme Pentose Phosphate Pathway Sedoheptulose 7-phosphate + D-glyceraldehyde 3-phosphate <=> D-ribose 5-phosphate + D-xylulose 5-phosphate	Transcriptional regulator derepresses the marRAB multiple antibiotic resistance operon by binding to the MarR repressor, a “trigger enzyme”	0
39	KAA8472657.1	Glutamate-tRNA ligase	Glutamyl-prolyl tRNA synthetase, *Homo sapiens*	Glutamyl-prolyl tRNA synthetase, enzyme an aminoacyl-tRNA synthetase catalyze the attachment of amino acids to cognate tRNAs ATP + L-glutamate + tRNA(Glu) => AMP + diphosphate + L-glutamyl-tRNA(Glu) ATP + L-proline + tRNA(Pro) => AMP + diphosphate + L-prolyl-tRNA(Pro)	Translation inhibition part of GAIT complex: interferon (IFN)-gamma-activated inhibitor of translation silences ceruloplasmin mRNA translation	1.85 × 10^−15^
40	KAA8472519.1	HtrA protease/chaperone protein	DegP, *Escherichia coli*	Peptidase at higher temperatures	Chaperone at low temperatures	3.81 × 10^−72^
41	KAA8472533.1	Hihydrolipoamide succinyltransferase component (E2) of 2- oxoglutarate dehydrogenase complex	Alpha-ketoglutarate dehydrogenase E2, *Trypanosoma brucei*	Alpha-ketoglutarate dehydrogenase E2, in Krebs cycle, dihydrolipoyl succinyltransferase	Mitochondrial DNA inheritance	1.24 × 10^−87^
42	KAA8472539.1	Metalloprotease	DegP, *Escherichia coli*	Peptidase at higher temperatures.	Chaperone at low temperatures	9.6 × 10^−7^
43	KAA8472556.1	ATP-dependent metallopeptidase FtsH/Yme1/Tma family protein	FtsH, *Shigella flexneri*	Chaperone	Metalloprotease, enzyme ATP-dependent zinc metallopeptidase, hydrolyzes cytoplasmic and transmembrane proteins	0
44	KAA8472563.1	Isocitrate dehydrogenase	Isocitrate dehydrogenase 2, *Saccharomyces cerevisiae*	Isocitrate dehydrogenase, enzyme isocitrate + NAD^+^ = 2-oxoglutarate + CO_2_ + NADH Citric acid cycle	Binds mRNA specifically to 5′-untranslated leaders of mitochondrial mRNAs	6.43 × 10^−74^
45	KAA8472473.1	Hsp70 family protein	DnaK, *Neisseria meningitidis*	Chaperone	Plasminogen binding protein	4.09 × 10^−106^
46	KAA8472398.1	50S ribosomal protein L1	L1 ribosomal protein, *Escherichia coli*	Ribosomal protein, part of the 50S subunit	Translational repressor binds to the mRNA of the L11 operon	2.37 × 10^−56^
47	KAA8472399.1	50S ribosomal protein L10	L10 ribosomal protein, *Escherichia coli*	Ribosomal protein part of the 50S subunit	Translation inhibitor autogenous regulation of translation	6.83 × 10^−10^
48	KAA8472401.1	DNA-directed RNA polymerase subunit beta	DNA-directed RNA polymerase beta subunit, *Streptococcus gordonii*	Beta subunit of DNA-directed RNA polymerase	Muc7 binding protein	7.26 × 10^−41^
49	KAA8472421.1	Succinate dehydrogenase, cytochrome b556 subunit	Succinate dehydrogenase subunit 3, *Saccharomyces cerevisiae*	Subunit of succinate dehydrogenase in the respiratory chain, electron transport in respiratory complex II	Part of TIM22 complex (carrier translocase, mitochondrial inner membrane translocase) in mitochondria, helps in biogenesis and assembly of membrane-integral subunits of TIM22 complex	2.33 × 10^−9^
50	KAA8472432.1	ATP-dependent Clp protease ATP-binding subunit ClpX	FtsH, *Shigella flexneri*	Chaperone	Metalloprotease, enzyme ATP-dependent zinc metallopeptidase, hydrolyzes cytoplasmic and transmembrane proteins	6.18 × 10^−7^
51	KAA8472433.1	Endopeptidase La	FtsH, *Shigella flexneri*	Chaperone	Metalloprotease, enzyme ATP-dependent zinc metallopeptidase, hydrolyzes cytoplasmic and transmembrane proteins	8.2 × 10^−9^
52	KAA8472438.1	30S ribosomal protein S4	S4 ribosomal protein, *Escherichia coli*	S4 ribosomal protein part of the 30S subunit, helps nucleate assembly of the 30S subunit by binding directly to the 16S rRNA	Translational repressor binds mRNA of operon encoding S3, S11, S4	6.05 × 10^−41^
53	KAA8472355.1	Isoleucine-tRNA ligase	Leucyl-tRNA synthetase, *Saccharomyces cerevisiae*	Leucyl-tRNA synthetase, enzyme ATP + L-leucine + tRNA (Leu) => AMP + diphosphate + L-leucyl-tRNA (Leu) protein synthesis	Intron splicing, RNA splicing group I intron splicing	4.64 × 10^−14^
54	KAA8472356.1	Thiol peroxidase (Peroxiredoxin)	Peroxiredoxin, *Neisseria meningitidis*	Peroxiredoxin, antioxidant	Plasminogen binding	1.29 × 10^−34^
55	KAA8472364.1	NADH-quinone oxidoreductase subunit NuoI	Pyruvate-ferredoxin oxidoreductase, *Trichomonas vaginalis*	Pyruvate-ferredoxin oxidoreductase, enzyme oxidative decarboxylation of pyruvate to yield acetyl-CoA and CO_2_	Cell surface adherence protein	9.65 × 10^−5^
56	KAA8472372.1	Translational GTPase TypA	EF-G, *Streptococcus gordonii*	Elongation factor in translation catalyzes translocation step, uses GTP	Adhesin, binds salivary mucin MUC7	7.93 × 10^−27^
57	KAA8472305.1	Pyruvate dehydrogenase complex E1 component subunit beta	Pyruvate dehydrogenase, *Mycoplasma pneumoniae*	Pyruvate dehydrogenase, enzyme, the pyruvate dehydrogenase complex catalyzes the overall conversion pyruvate => acetyl-CoA and CO_2_. Pyruvate dehydrogenase (E1) is one of the enzyme components of the complex	Fibrinogen binding	1.09 × 10^−56^
58	KAA8472326.1	Lipoprotein releasing (ABC transporter ATP-binding protein)	MalK, *Escherichia coli*	ATP binding/hydrolysis protein of MalEFGK maltose/maltodextrin transporter (importer) an ABC transporter ATP + H_2_O = ADP + phosphate	Transcription regulation binds to MalT activator of mal regulon and prevents its action	8.20 × 10^−23^
59	KAA8472251.1	Holliday junction branch migration DNA helicase RuvB	Regulatory particle triple-A ATPase subunit 5b, *Arabidopsis thaliana*	Part of the regulatory ATPase complex of the 26S proteasome, confers substrate specificity and need for ATP to the proteasome	Binds to hexokinase 1 and VHA B1 to modulate transcription of specific target genes	3.89 × 10^−6^
60	KAA8472256.1	ABC transporter (ATP-binding cassette domain-containing protein)	MDR1, *Homo sapiens*	Transmembrane transporter efflux pump, uses ATP for energy expels drugs and other small molecule compounds ATP + H_2_O + xenobiotic (Inside) => ADP + phosphate + xenobiotic (Outside)	Regulator of volume-activated chloride channels	6.69 × 10^−79^
61	KAA8472258.1	Serine hydroxymethyltransferase	Serine hydroxymethyltransferase, *Homo sapiens*	Serine hydroxymethyltransferase, enzyme 5,10-methylenetetrahydrofolate + glycine + H_2_O <=> tetrahydrofolate + L-serine one-carbon metabolism, tetrahydrofolate interconversion	Binds mRNA binds the 5′ untranslated region (UTR) of its own mRNA	1.29 × 10^−103^
62	KAA8472274.1	Heme ABC exporter ATP-binding protein CcmA	MalK, *Escherichia coli*	ATP binding/hydrolysis protein of MalEFGK maltose/maltodextrin transporter (importer) an ABC transporter ATP + H_2_O = ADP + phosphate	Transcription regulation binds to MalT activator of mal regulon and prevents its action	1.29 × 10^−14^
63	KAA8472191.1	50S ribosomal protein L4	L4 ribosomal protein, *Escherichia coli*	Ribosomal protein, part of the 50S subunit	Transcriptional repressor causes premature termination of transcription within S10 operon	2.13 × 10^−26^
64	KAA8472193.1	50S ribosomal protein L2	L2 ribosomal protein, *Saccharomyces cerevisiae*	Component of the ribosome large subunit (60S)	Regulates accumulation of L2 mRNA, shortens half-life of L2 mRNA	2.66 × 10^−24^
65	KAA8472200.1	50S ribosomal protein L14	L14 ribosomal protein, *Escherichia coli*	Ribosomal protein part of the 30S subunit binds to the 23S rRNA	Binds DNA, stimulates unwinding of DNA by Rep helicase protein	3.07 × 10^−40^
66	KAA8472202.1	50S ribosomal protein L5	L11 ribosomal protein, *Mus musculus*	Ribosomal protein, part of 60S subunit	Binds to and inhibits HDM2, a ubiquitin ligase, which results in stabilization of p53 tumor suppressor protein	1.31 × 10^−13^
67	KAA8472204.1	30S ribosomal protein S8	S8 ribosomal protein, *Escherichia coli*	S8 ribosomal protein part of the 30S subunit binds to 16S rRNA	Translational repressor inhibits expression of some proteins encoded by the spc operon	7.13 × 10^−41^
68	KAA8472212.1	30S ribosomal protein S11	S14 ribosomal protein, *Saccharomyces cerevisiae*	Component of the ribosome small subunit (40S)	Represses expression of RPS14B gene, rpS14 binds directly to RNA, binds to an RNA stem-loop structure in RPS14B pre-mRNA	1.5 × 10^−9^
69	KAA8472223.1	Phosphate ABC transporter ATP-binding protein	MDR1, *Homo sapiens*	Transmembrane transporter efflux pump, uses ATP for energy, expels drugs and other small molecule compounds ATP + H_2_O + xenobiotic (Inside) => ADP + phosphate + xenobiotic (Outside)	Regulator of volume-activated chloride channels	6.61 × 10^−31^
70	KAA8472142.1	UMP kinase	PyrH, *Escherichia coli*	UMP kinase, enzyme phosphorylates UMP to UDP ATP + UMP <=> ADP + UDPde novo biosynthetic pathway of pyrimidine nucleotides	Transcriptional regulator involved in pyrimidine-specific repression of the carAB operon binds to PepA	1.55 × 10^−74^
71	KAA8472163.1	Thioredoxin	Thioredoxin, *Escherichia coli*	Thioredoxin antioxidant aids the reduction of other proteins by cysteine thiol-disulfide exchange	Subunit of T7 DNA polymerase (maybe not strictly considered moonlighting because adopted by a phage to be part of a protein complex)	6.18 × 10^−26^
72	KAA8472099.1	Glutamine synthetase	Glutamine synthetase, Bifidobacterium (*Bifidobacterium lactis*, *B. bifidum*, and *B. longum*)	Glutamine synthetase, enzyme ATP + L-glutamate + NH_3_ => ADP + phosphate + L-glutamine	Plasminogen binding	3.53 × 10^−16^
73	KAA8472120.1	30S ribosomal protein S7	S7 ribosomal protein, *Escherichia coli*	Ribosomal protein binds to 16S rRNA part of the 30S subunit	Translational repressor regulates the expression of two proteins encoded by the str operon	2.05 × 10^−46^
74	KAA8472121.1	Elongation factor G	EF-G, *Streptococcus gordonii*	Elongation factor in translation catalyzes translocation step, uses GTP	Adhesin, binds salivary mucin MUC7	0
75	KAA8471941.1	Elongation factor Tu	Translation elongation factor Tu, *Streptococcus gordonii*	Translation elongation factor Tu	Mucin (MUC7)-binding protein	3.29 × 10^−12^
76	KAA8472015.1	Aspartate tRNA ligase	Lysyl-tRNA synthetase, *Homo sapiens*	Lysyl-tRNA synthetase, enzyme ATP + L-lysine + tRNA (Lys) = AMP + diphosphate + L-lysyl-tRNA(Lys) protein synthesis	Cytokine binds to macrophages and peripheral blood mononuclear cells, increases TNF-alpha Production by target cells increases target cell migration	5.64 × 10^−14^
77	KAA8472021.1	Cytochrome c family protein	Cytochrome C, *Equus caballus*	Electron carrier protein component of the mitochondrial electron-transport chain	Binding to apoptosis protease activation factor-1 (Apaf-1), promotes apoptosis release from mitochondria, allows interaction with apoptosis proteins	2.04 × 10^−26^
78	KAA8471995.1	Bifunctional proline dehydrogenase/L-glutamate gamma-semialdehyde dehydrogenase	PutA, *Salmonella typhimurium*	Proline dehydrogenase/Proline oxidase pyrroline-5-carboxylic acid dehydrogenase activity	Transcriptional repressor of the put operon	0
79	KAA8472002.1	Phosphopyruvate hydratase	Enolase, *Bacillus anthracis*	Enolase, enzyme 2-phospho-D-glycerate => phosphoenolpyruvate + H_2_O, catalyzes the reversible conversion of 2-phosphoglycerate into phosphoenolpyruvate, carbohydrate degradation, glycolysis	Binds plasminogen and laminin	8.84 × 10^−171^
80	KAA8471950.1	Malate dehydrogenase	Lactate dehydrogenase isozyme M, *Mus musculus*	Enzyme, lactate + NAD^+^ -> pyruvate + NADH	RNA binding protein, post-transcriptional regulation of gene expression, AU-rich element (ARE, binding to these kinds of elements can be involved in affecting RNA stability) binding protein (AUBP), role may also involve binding to AUF1 protein, binds the 3-UTR of GM-CSF RNA	3.98 × 10^−52^

**Table 2:** B-cell epitopes predicted for *A. marginale* AmEno, AmGroEl, AmDnaK, and AmEF-Tu in BcePred and SVMTrip. The number in the parentheses corresponds to the score predicted for the SVMTrip epitope (the score closest to 1 represents the best prediction). The bold sequences correspond to B-cell epitopes used to design multiple antigenic peptides AmEno1 and AmEno2.

MLPs	B-Cell Epitopes	
	BCEPred	SVMTrip
AmEno	**YNVIISHRSGETEDVTIA** VSPFDQRAVDEILLSLDGTKNKSKLG YKLKEVLKKMGHSTNTGDEGGFA PNLENNTDVLDVLVEAIERSGYRASSDV	**DIEGWKAVTKRLGDKIQL** (1.0) CAEVFYKLKEVLKKMGHSTN (0.861) SYKFSGKCLTSGELIACYED (0.859)
AmEF-Tu	LLRGIKKEDVERGQVL GQIRSYKAFKAEVYILKKEEGGRHTPF KIELPVREKDKPFLM	SEKIMELVGALEKIELPVRE (1.0)
AmGroEl	QIKSQIEVSSSDYDKEKLKERLAKL QCVREVGKDGVITVEESK GFKDLEVERTDGMQF	KKINLVQSILPVLENVARSG (1.0) MANVVVTGEALDKSIREVVR (0.975) EDEIAQVATISANGDKNIGG (0.809)
AmDnaK	AFTENERLVGELAKRQANINAQNTIYASKRIIGRRYDDMRDVKCPY AKHLSLKLTRAKFEGLVSELIERTIEPCKKALDDAGIKDTSKIDE SDTSGNPEERVVDSEYQEIKKDDEDKK	GDKISSADKSGIEAAIKELR (1.0) VLEIAEGVFEVKATNGDTKL (0.999) GAKHLSLKLTRAKFEGLVSE (0.867)

## Data Availability

Data generated or analyzed during this study are available in the published article.

## Supplementary Materials

The following supporting information can be downloaded at: https://www.mdpi.com/article/10.3390/pathogens13100845/s1, Table S1: Name and functional classification of *A. marginale* strain MEX-15-099-01 MLPs.
