# Supplementary material for: Molecular Identification and Bioinformatics Analysis of Anaplasma marginale Moonlighting Proteins as Possible Antigenic Targets
Source: Pathogens. 2024 Sep 28;13(10):845. doi: 10.3390/pathogens13100845 (PMC11510912; doi:10.3390/pathogens13100845)
Supplement: Supplementary file 1 [file pathogens-13-00845-s001.zip › pathogens-3171106-supplementary.pdf]

Supplementary Table S1. Name and functional classification of *A. marginale* strain MEX-15-099-01 MLPs.

	**NCBI ID**	**RAST annotation of *A. marginale* strain MEX-15-099-01 MLPs**	**COG Functional group**	**COG Gene Name**
1	KAA8473050.1	DNA mismatch repair endonuclease MutL	L	*mutL*
2	KAA8473081.1	ABC transporter ATP-binding protein	Q	*mkL*
3	KAA8473084.1	Biotin-[acetyl-CoA-carboxylase] ligase	H	*birA*
4	KAA8473103.1	Porphobilinogen synthase	H	*hemC*
5	KAA8473122.1	Leucine-tRNA ligase	J	*leuS*
6	KAA8473129.1	excinuclease ABC subunit UvrA	L	*uvrA*
7	KAA8473164.1	Proline-tRNA ligase	J	*proS*
8	KAA8473168.1	Peroxiredoxin	O	*prdx1*
9	KAA8473177.1	Translation elongation factor LepA	J	*lepA*
10	KAA8473193.1	Triosephosphate isomerase	G	*tpiA*
11	KAA8473199.1	Adenylosuccinate lyase	F	*purB*
12	KAA8473204.1	Pyridoxal phosphate- dependent aminotransferase	H	*bioA*
13	KAA8473206.1	Rod shape-determining protein	D	*mreB*
14	KAA8472839.1	Phosphopantothenoylcysteine decarboxylase	H	*coaD*
15	KAA8472842.1	Phosphoglycerate kinase	F	*Pgk*
16	KAA8472854.1	Ketol-acid reductoisomerase	H	*Ilvc*
17	KAA8472865.1	Molecular chaperone DnaJ	A	*dnaJ*
18	KAA8472874.1	Type I glyceraldehyde-3-phosphate dehydrogenase	G	*Gap*
19	KAA8472877.1	Aspartate-semialdehyde dehydrogenase	E	*Asd*
20	KAA8472881.1	Threonine-tRNA ligase	J	*thrS*
21	KAA8472917.1	Ornithine carbamoyltransferase	E	*argF*

22	KAA8472922.1	ATP-binding cassette domain-containing protein	V	*msbA2*
23	KAA8472925.1	Glutamine synthetase	E	*glnA*
24	KAA8472933.1	Pyruvate dehydrogenase complex dihydrolipoamide acetyltransferase	C	*pdhB*
25	KAA8472947.1	Valine-tRNA ligase	J	*ileS*
26	KAA8472967.1	Tyrosine-tRNA ligase	J	*tyrS*
27	KAA8472719.1	Quinone oxidoreductase	C	*Qor*
28	KAA8472720.1	Type I secretion system permease/ATPase	U	*yidC*
29	KAA8472725.1	Class II fumarate hydratase	C	*fumC*
30	KAA8472731.1	Chaperonin GroEL	O	*Groel*
31	KAA8472736.1	Cytosol aminopeptidase PepA	E	*pepA*
32	KAA8472754.1	NAD(P)H-dependent glycerol-3-phosphate dehydrogenase	I	*gpsA*
33	KAA8472760.1	ATP-dependent protease ATPase subunit HslU	O	*hslU*
34	KAA8472784.1	Aconitate hydratase AcnA	C	*AcnA*
	KAA8472802.1	Methionine-tRNA ligase	J	*infB*
36	KAA8472628.1	Superoxide dismutase	O	*tdPX1*
37	KAA8472646.1	Molecular chaperone DnaK	O	*dnaK*
38	KAA8472652.1	Transketolase	G	*Tkt*
39	KAA8472657.1	Glutamate-tRNA ligase	J	*gatC*
40	KAA8472519.1	HtrA protease/chaperone protein	M	*htrA*
41	KAA8472533.1	Hihydrolipoamide succinyltransferase component (E2) of 2- oxoglutarate dehydrogenase complex	C	*sucB*
42	KAA8472539.1	Metalloprotease	C	*Fdxb*
43	KAA8472556.1	ATP-dependent metallopeptidase FtsH/Yme1/Tma family protein	O	*ftsH*
44	KAA8472563.1	Isocitrate dehydrogenase	C	*Icd*

45	KAA8472473.1	Hsp70 chaperone family protein HscA	O	*hscA*
46	KAA8472398.1	50S ribosomal protein L1	J	*rpIA*
47	KAA8472399.1	50S ribosomal protein L10	J	*rpIJ*
48	KAA8472401.1	DNA-directed RNA polymerase subunit beta	K	*rpoB*
49	KAA8472421.1	Succinate dehydrogenase, cytochrome b556 subunit	C	*sdhC*
50	KAA8472432.1	ATP-dependent Clp protease ATP-binding subunit ClpX	O	*Clpx*
51	KAA8472433.1	Endopeptidase La	Q	
52	KAA8472438.1	30S ribosomal protein S4	J	*rpsD*
53	KAA8472355.1	Isoleucine-tRNA ligase	J	*tilS*
54	KAA8472356.1	Thiol peroxidase (Peroxiredoxin)	O	*Bcp*
55	KAA8472364.1	NADH-quinone oxidoreductase subunit NuoI	C	*ma20*
56	KAA8472372.1	Translational GTPase TypA	T	*typA*
57	KAA8472305.1	Pyruvate dehydrogenase complex E1 component subunit beta	C	*pdhA*
58	KAA8472326.1	Lipoprotein releasing (ABC transporter ATP-binding protein)	V	*lolD*
59	KAA8472251.1	Holliday junction branch migration DNA helicase RuvB	L	*ruvB*
60	KAA8472256.1	ABC transporter (ATP-binding cassette domain-containing protein)	G	*tpiA*
61	KAA8472258.1	Serine hydroxymethyltransferase	E	*glyA*
62	KAA8472274.1	Heme ABC exporter ATP-binding protein CcmA	P	*ccmA*
63	KAA8472191.1	50S ribosomal protein L4	J	*rpID*
64	KAA8472193.1	50S ribosomal protein L2	J	*rpIB*
65	KAA8472200.1	50S ribosomal protein L14	J	*rpIN*
66	KAA8472202.1	50S ribosomal protein L5	J	*rpIE*
67	KAA8472204.1	30S ribosomal protein S8	J	*rpsH*
68	KAA8472212.1	30S ribosomal protein S11	J	*rpsK*

69	KAA8472223.1	Phosphate ABC transporter ATP-binding protein	J	*pstB*
70	KAA8472142.1	UMP kinase	P	*pyrH*
71	KAA8472163.1	Thioredoxin	F	*Txn*
72	KAA8472099.1	Glutamine synthetase	O	*pdhC*
73	KAA8472120.1	30S ribosomal protein S7	C	*rpsG*
74	KAA8472121.1	Elongation factor G	J	*fusA*
75	KAA8471941.1	Elongation factor Tu	J	*Tst*
76	KAA8472015.1	Aspartate tRNA ligase	J	*aspS*
77	KAA8472021.1	Cytochrome c family protein	C	*sodB*
78	KAA8471995.1	Bifunctional proline dehydrogenase/L-glutamate gamma-semialdehyde dehydrogenase	E	*putA*
79	KAA8472002.1	Phosphopyruvate hydratase (enolase)	F	*Eno*
80	KAA8471950.1	Malate dehydrogenase	C	*Mdh*
